# Acquiring Process Knowledge in Extrusion-Based Additive Manufacturing via Interpretable Machine Learning

**DOI:** 10.3390/polym15173509

**Published:** 2023-08-23

**Authors:** Lukas Pelzer, Tobias Schulze, Daniel Buschmann, Chrismarie Enslin, Robert Schmitt, Christian Hopmann

**Affiliations:** 1 Institute for Plastics Processing at RWTH Aachen University, 52074 Aachen, Germany; 2 Laboratory for Machine Tools and Production Engineering, RWTH Aachen University, 52074 Aachen, Germany; t.schulze@wzl-mq.rwth-aachen.de (T.S.); d.buschmann@wzl-mq.rwth-aachen.de (D.B.);; 3 Cybernetics Lab IMA & IfU, RWTH Aachen University, 52068 Aachen, Germany

**Keywords:** additive manufacturing, feature importance, fused layer modeling, interpretable machine learning, machine Learning, process characterization, process knowledge, process optimization

## Abstract

Additive manufacturing (AM), especially the extrusion-based process, has many process parameters which influence the resulting part properties. Those parameters have complex interdependencies and are therefore difficult if not impossible to model analytically. Machine learning (ML) is a promising approach to find suitable combinations of process parameters for manufacturing a part with desired properties without having to analytically model the process in its entirety. However, ML-based approaches are typically black box models. Therefore, it is difficult to verify their output and to derive process knowledge from such approaches. This study uses interpretable machine learning methods to derive process knowledge from interpreted data sets by analyzing the model’s feature importance. Using fused layer modeling (FLM) as an exemplary manufacturing technology, it is shown that the process can be characterized entirely. Therefore, sweet spots for process parameters can be determined objectively. Additionally, interactions between parameters are discovered, and the basis for further investigations is established.

## 1. Introduction

Using additive manufacturing (AM), a complex object can be created layer by layer without the need for part-specific tooling or extensive machine set-ups [1]. This way, geometries can be produced that would otherwise be impossible to manufacture, like parts with complex internal cavities or complete assemblies [2]. Via AM, a large number of different materials for various use-cases can be processed [3]. However, the technology’s large degree of freedom is accompanied by its complexity. Historically, the technology has mostly been used for prototyping and small-scale production runs [4]. Considering the technology’s benefits, it is well suited for decentralized production [5]. Therefore, AM has the potential to address many current socio-economic challenges like part shortages as a result of a global pandemic or geopolitical conflicts and global CO_2_-emissions as a result of shipping parts across the globe. However, to transform AM into a widely accessible technology, it has to be easy to use and part quality has to be consistent regardless of production location. To achieve this, the process and the quality-defining parameters and interdependencies have to be well understood.

Globally, the most-used AM technology is fused layer modeling (FLM; also fused deposition modeling—FDM) [4]. While offering many benefits and great freedom in part design, such as a wide material spectrum as well as material and time savings by using sparse infill patterns, path planning software (slicing software) for FLM offers a large number of process parameters which have to be set. The technology’s complexity comes from the number of process parameters that can be adjusted, their influence on part quality, as well as the interdependencies between those process parameters. In most slicers, hundreds of parameters can be set. According to [6], the most impactful parameters are the following:

- Layer thickness
- Build orientation
- Print speed *
- Raster width *
- Nozzle temperature
- Infill density
- Air gap *
- Raster orientation *
- Infill pattern
- Number of shells
- Shell width

However, ref. [6] also notes that the significance of some parameters regarding certain quality criteria is yet to be determined (indicated with *). It is important to understand the parameters and their effects and interdependencies to produce parts that meet requirements, e.g., regarding mechanical properties, dimensional accuracy, surface quality, etc., without extensive trials. This is especially relevant for small-scale production runs or lot-size-one production. Furthermore, a better understanding of correlations is beneficial for improving the technology by understanding its limitations. It can be used to increase manufacturing speed while knowing that quality criteria are still being met. Lastly, a good understanding of the process provides the necessary basis for simulation routines, which are not yet widely developed for FLM. This is mostly due to the complexity of the modeling task, which is a multi-scale, multi-physics endeavor with complex interactions between algorithms [7].

To contribute to the understanding of the process parameters in FLM, their effects on part quality as well as their interdependencies, this study investigates the FLM-technology’s parameters of nozzle temperature, manufacturing speed, part cooling and part orientation, their effects on part quality and their interdependencies by training machine learning (ML) models and interpreting the learned characteristics of the underlying process from the data afterwards.

Especially for production problems with complex interactions, these are difficult or impossible to capture with analytical models or inherently interpretable ML models such as linear regression models or decision trees [8]. More complex modeling approaches offer the potential to learn and describe the process behavior. Nevertheless, since in most cases the latter are black box models, they are no longer inherently interpretable [8]. This prevents the extraction of knowledge from the considered models that would enhance the understanding of the process and ultimately contribute to the optimization of the manufacturing process. At this point, methods of interpretable ML can be used to show the effects of the input variables and their interactions on which the output of the inherently uninterpretable model is based and to derive knowledge about the underlying process.

## 2. State of the Art

### 2.1. Influence of Process Parameters in Fused Layer Modeling

The process parameters investigated in this study, i.e., nozzle temperature, part cooling, manufacturing speed and part orientation, have been chosen because they exhibit interdependencies regarding the considered quality criteria, i.e., tensile strength, Young’s modulus, dimensional accuracy, weight accuracy and manufacturing time.

Increasing nozzle temperature typically leads to improved mechanical properties, namely, tensile strength and Young’s modulus [9,10]. However, it has to be noted that increasing temperatures beyond the degradation temperature of the polymer has a detrimental effect. While generally improving mechanical properties, increased nozzle temperature negatively impacts dimensional accuracy since the material remains in a molten state for longer [11]. Regarding weight accuracy, increased nozzle temperature is more likely to plasticize and therefore extrude the demanded amount of material, which is why a positive effect can typically be expected [12].

The effects of part cooling, which is used to blow cold air on the extruded strands just after deposition to support cooling and hardening of the strands to better support forthcoming layers, are generally inverse to nozzle temperature. Increased part cooling improves dimensional accuracy since the material is more quickly solidified in its intended position. At the same time, part cooling reduces tensile strength and Young’s modulus since the contact temperature between previous and current strands are reduced, reducing the strength of the weld line [13]. Neither nozzle temperature nor part cooling affect manufacturing time, which is, however, greatly impacted by manufacturing speed.

Increasing manufacturing speed lowers the total manufacturing time [6]. Adjusting this process parameter has complex interdependencies with nozzle temperature and part cooling, since a faster moving machine also has to plasticize material more quickly. At the same time, individual strands have less time to cool before the next layer is applied. Therefore, increasing manufacturing speed also has to be coupled with a careful balancing of increased nozzle temperature and increased part cooling. If those parameters are not adjusted, the mechanical properties and dimensional accuracy will deteriorate with increased manufacturing speed [14]. Also, weight accuracy is likely to be negatively affected since less material can be plasticized.

Since FLM is a layer-based technology, part orientation has a major impact on tensile strength and Young’s modulus [15]. Studies have found that loading a part in the z direction (build direction) can reduce its tensile strength to 50% or less compared to a part loaded in the x or y direction (parallel to the build surface) [16]. At the same time, part orientation influences dimensional accuracy and manufacturing time [17].

### 2.2. Determining Feature Importance with Interpretable Machine Learning

To evaluate these literature-based findings regarding the influence of process parameters on part quality in the FLM processes, ML models are trained and analyzed with interpretable ML methods.

Interpretable ML is concerned with extracting relevant knowledge from ML models in terms of which features are important and which interactions exist between them. Since the technique works on the model and not directly on the data, interpretable ML can only identify the information on which the model bases its decision. Only if the model correctly captures the relationships within the data is it possible to derive insights about the information contained in the data [18].

There are plenty of different methods, each extracting a certain kind of information from the model. For example, it is possible to derive whether a feature is important for the model prediction with some methods. Others can depict how certain feature values influence this prediction. The real strength lies in cleverly combining these methods to derive more comprehensive insights from the model [19].

Within this publication Permutation Feature Importance (PFI) and Accumulated Local Effects (ALE) are used for this purpose. Firstly, PFI is a condensed statistical value per feature stating its importance for the model prediction. To calculate this value, for the different datapoints the value of the given feature is permuted over its value range while the values of the other features are kept. Followingly, the errors of the predictions of the original and permuted datapoints are compared, resulting in the importance value. The bigger the impact of the permutation on the model error, the higher the impact of the feature on the model prediction will be, leading to a higher feature importance value [20].

**Figure 1:**
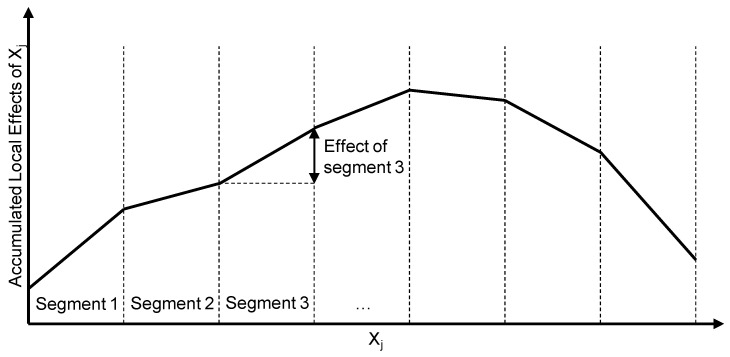
Accumulated local effects of feature Xj. For each segment of Xj’s value range, the local effects are calculated and accumulated afterwards.

Secondly, ALE are used to look more closely at the features that are important according to PFI. They depict the effect of a feature value on the model prediction. For calculation, the data set is divided into different segments according to the value of the considered feature. For each data point in a segment, the value of the considered feature is set once to the left and once to the right boundary of the interval, while the values for the other features are maintained. For these adjusted data points, the model prediction is calculated. Then, the difference between the two model predictions is formed for each data point, and, finally, these differences are averaged. For the respective effect of a concrete value of the considered input variable, the results of all segments are added up to this point (see Figure 1) [21].

## 3. Materials and Methods

### 3.1. Trials for Data Generation

To set up a data basis for the feature importance and feature interaction evaluation of FLM parameters, trials were conducted. For this, tensile test specimens according to DIN EN ISO 527 [22], specimen type 1BA, were manufactured on an Ender 3 FLM 3D printer by manufacturer Creality 3D Technology Co., Ltd. (Shenzhen, China) from natural polylactic acid (PLA) from manufacturer Fillamentum Manufacturing Czech s.r.o., Hulín, Czech Republic. G-code was prepared using the slicer Simplify3D from the company Simplify3D, Cincinnati, OH, USA. For the trials, process parameters were chosen according to Table 1. Here, the varied parameters were varied in five equidistant steps with the exception of part orientation, which was treated as a binary parameter with the manifestations lying (manufactured in the x-y plane) and standing (manufactured in the y-z plane).

Regarding the experiment’s design, a central composite design was chosen for the three parameters of nozzle temperature, part cooling and manufacturing speed. The resulting trial plan represents a three-dimensional set-up in the shape of a star pattern around a central point. Additionally, the extreme points (corner points) were tested. To avoid polluting the results with outliers, five lying and five standing samples were manufactured per trial point.

After manufacturing, each sample was evaluated regarding its tensile strength, Young’s modulus, elongation at tensile strength, manufacturing time, dimensional accuracy and weight accuracy. Mechanical results were obtained by using the measured cross-section of each individual specimen (three measurements, averaged) instead of the measurements as designed, to account for slight variations in manufacturing and improve result accuracy. Weight accuracy was calculated by comparing the measured weight of each specimen to the target weight as calculated by the slicer. The results are shown in Table 2. The trial designation represents the varied parameters: nozzle temperature—manufacturing speed—part cooling—orientation.

### 3.2. Data Analysis Pipeline to Determine Feature Importance in Fused Layer Modeling

**Figure 2:**
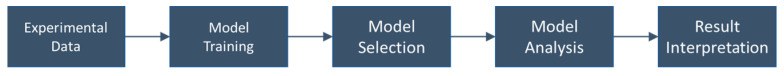
Data analysis pipeline to determine feature importance.

For the analysis of the process via the interpretable machine learning methods, ML models are first required, which are trained via the generated test data (see previous section) and are provided for further evaluation (Figure 2). For this purpose, different ML models are first trained, evaluated and then selected based on their model performance. The chosen ML models are then analyzed using ALE and PFI (see Section 2), and the results are interpreted and compared with the physical expectations.

**Figure 3:**
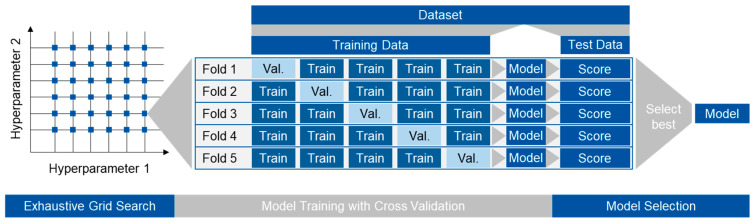
For each point in the grid set up by the hyperparameters (here: maximal depth of tree, minimal samples per split, minimal samples per leaf and, depending on the model type, number of estimators), five-fold cross-validation is performed by splitting the training data into five parts. Five different models are trained using a different part of the training data for validation (val.). Based on the test data, the models are evaluated, and the model with the best score is selected.

As model types, decision tree regressor [23], extra tree regressor [24], random forest regressor [20] and gradient boosting regressor [25] are considered. For model training, 85% of the data were used. The remaining 15% were used for testing and result interpretation. To obtain the best performing models, an exhaustive grid search is performed using five-fold cross-validation on each parameter combination. Figure 3 depicts the approach that is performed for each model type and target. The parameters subject to optimization as well as their tested values are given in Table 3.

For each target, the best performing model according to the *R*^2^ score after hyperparameter optimization is selected. The results are shown in Table 4. While the models for Young’s modulus ($R^{2}=0.588$) and weight accuracy ($R^{2}=0.461$) show mediocre performance, the models perform quite well for the other targets ($R^{2}>0.900$). For the different target parameters, different model types perform best. To analyze these different models, it is necessary to use evaluation methods that are model-agnostic.

## 4. Results and Discussion

With the application of the model-agnostic PFI and ALE scores to the acquired data set in Table 2, the process can be analyzed in depth, and relationships and dependencies between process parameters and measured part properties can be investigated easily. The following paragraphs list the relevant findings as well as further investigations derived from the insights gained while using the feature importance toolkit.

### 4.1. General

**Figure 4:**
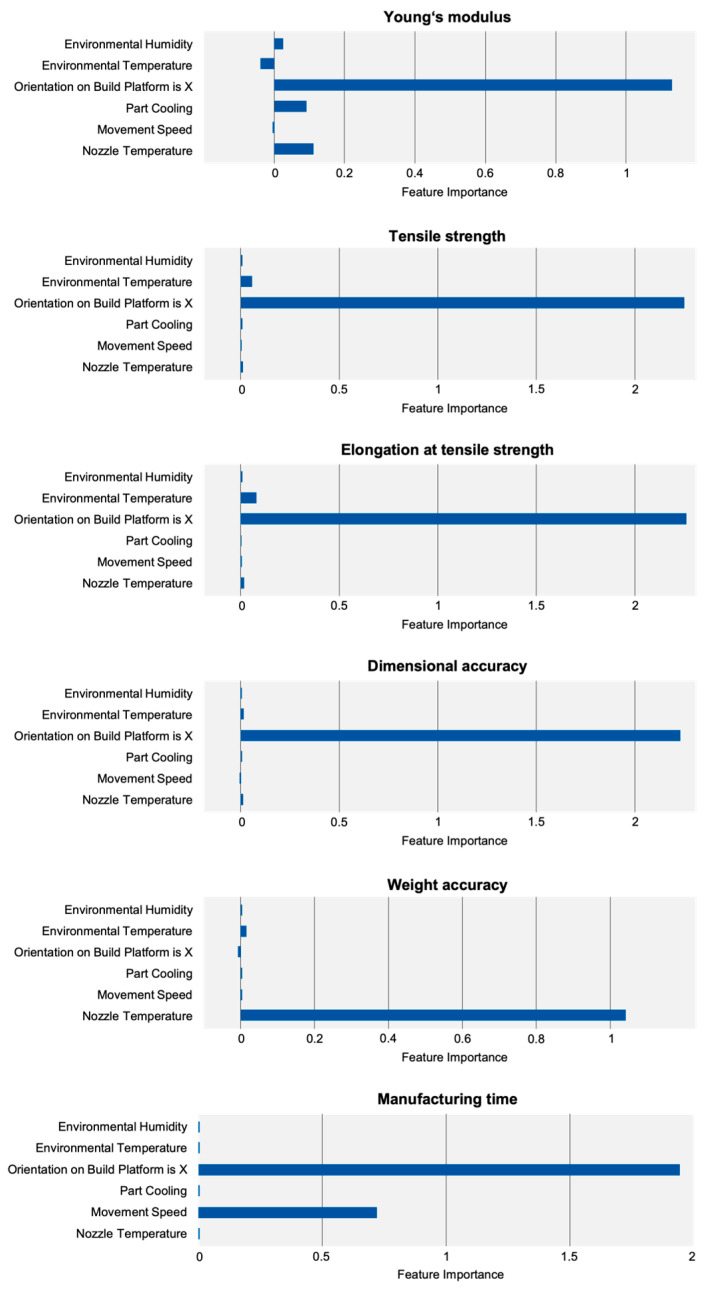
PFI scores indicating the importance of the process parameters (environmental humidity, environmental temperature, orientation on build platform, part cooling, movement speed and nozzle temperature) on the target values (Youngs’s modulus, tensile strength, elongation at tensile strength, dimensional accuracy, weight accuracy, manufacturing time).

Comparing all PFI scores (see Figure 4) shows that part orientation on the build plate is the most important factor out of all measured properties with the exception of weight accuracy. The latter mostly depends on nozzle temperature. This shows that, when optimizing for a real-world scenario where mechanical properties and dimensional accuracy are most important, part orientation and therefore strand deposition strategy has the largest impact. However, since part orientation is oftentimes dictated by part geometry and can rarely be freely chosen [26], it is also necessary to discuss the effects of other, more influenceable process parameters.

### 4.2. Young’s Modulus

The PFI score shows that Young’s modulus, like most part properties, is mostly dependent on part orientation. The three next highest-ranking parameters all have an impact on material temperature: environmental temperature, part cooling and nozzle temperature. Here, it is noticeable that nozzle temperature has a comparatively lower effect, with part cooling having a greater impact on the part stiffness. It has to be noted that the coefficient of determination for the Young’s modulus model is $R^{2}=0.558$. Therefore, absolute impact cannot be derived from small differences in the individual PFI scores.

**Figure 5:**
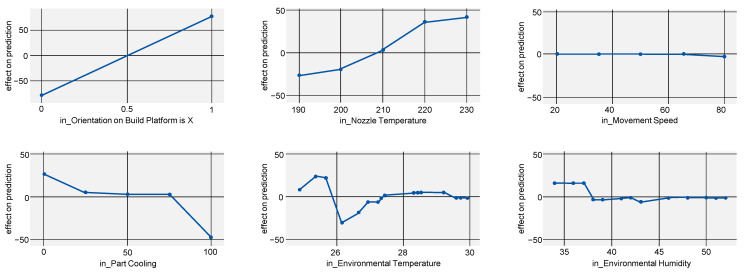
ALE indicating the effects of Young’s modulus on the model prediction when varying all investigated process parameters.

To not only show the absolute impact of a process parameter but also at which exact values part properties are improved, the ALE score can be regarded (see Figure 5). For Young’s modulus, this mostly proves the existing state of research: parts manufactured in lying orientation, a higher nozzle temperature and lower part cooling improve Young’s modulus. However, it is noticeable that the ALE for part cooling shows a flat curve with a steep decrease after 75% cooling, indicating this value as a candidate for a sweet spot.

Regarding the effects of environmental temperature and environmental humidity, it must be noted that the model used (gradient boosting regressor) is likely to overfit the data. This might explain the unclear progression of the curve.

### 4.3. Tensile Strength and Elongation at Tensile Strength

For tensile strength, the importance of part orientation is even more pronounced. With $R^{2}=0.939$, the derived findings are reliable.

**Figure 6:**
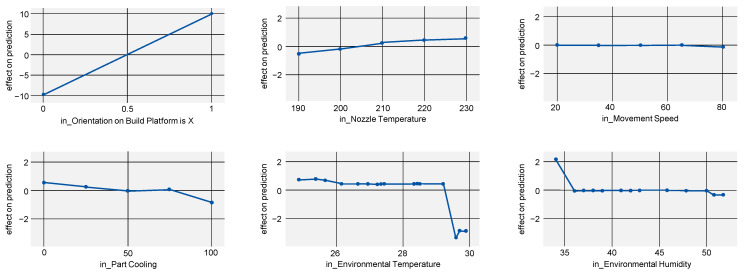
ALE indicating the effects of tensile strength on the model prediction when varying all investigated process parameters.

The curves for ALE, shown in Figure 6, are mostly flat, indicating further that parameters other than part orientation have no significant impact. Only for part cooling can the same decrease after 75% cooling as for Young’s modulus be observed, further reinforcing this value as a sweet spot.

**Figure 7:**
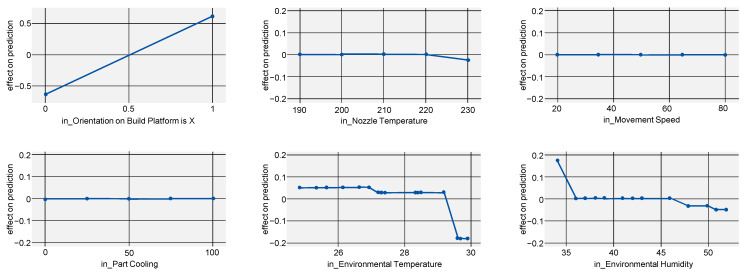
ALE indicating the effects of elongation at tensile strength on the model prediction when varying all investigated process parameters.

For elongation at tensile strength ($R^{2}=0.950$), the dependence on part orientation is even more pronounced, with other parameters having virtually no impact (see Figure 7).

### 4.4. Dimensional Accuracy

**Figure 8:**
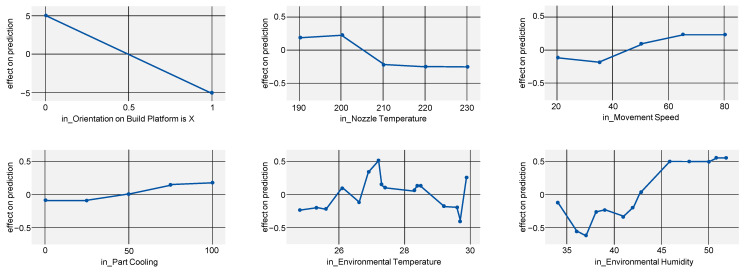
ALE indicating the effects of dimensional accuracy on the model prediction when varying all investigated process parameters.

ALE plots for dimensional accuracy (Figure 8, $R^{2}=0.943$) mainly support previous knowledge. A higher nozzle temperature decreases dimensional accuracy, while higher part cooling increases the value. This is typically explained by the time needed for the plasticized strand to solidify and retain its intended shape [13]. The lower the initial nozzle and therefore melt temperature and the higher part cooling is set, the faster an extruded strand can be cooled and solidified, preventing it from losing its shape or location.

With little benefit with regard to dimensional accuracy for part cooling beyond 75% (Figure 8, bottom left) but with a decrease in tensile strength and Young’s modulus (compare Figure 5, bottom left and Figure 6, bottom left), the selection of 75% part cooling as the optimal process point is further reinforced.

Just as with the ALE for Young’s modulus, it is possible that the model is overfitting the data for environmental temperature and humidity, which would explain their inconclusive results.

### 4.5. Weight Accuracy

Weight accuracy is the only part property where the PFI score indicates that nozzle temperature is the most influential process parameter. With $R^{2}=0.461$, the coefficient of determination is comparatively low. However, as the following investigations show, information derived from the feature importance toolkit is still valid and coincides with the real-world behavior of the system.

**Figure 9:**
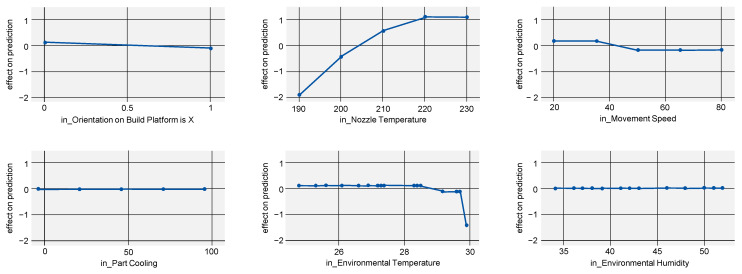
ALE indicating the effects of weight accuracy on the model prediction when varying all investigated process parameters.

Regarding the ALE scores shown in Figure 9, the expected positive correlation between a higher nozzle temperature and a higher weight accuracy can be observed. In combination with a slightly decreasing curve for movement speed, it can be assumed that the machine struggles to extrude the demanded amount of material for lower nozzle temperatures or higher speeds.

To investigate this relation further, the volumetric extrusion speed of the used extruder and plasticizing unit is measured. By extruding 200 mm of filament at flow rates between 2 mm^3^/s and 8 mm^3^/s and at temperatures between 190 °C and 230 °C and measuring the individual extrusions using a precision scale, the actual flow can be compared to the requested flow, and underextrusion can be determined. 

**Figure 10:**
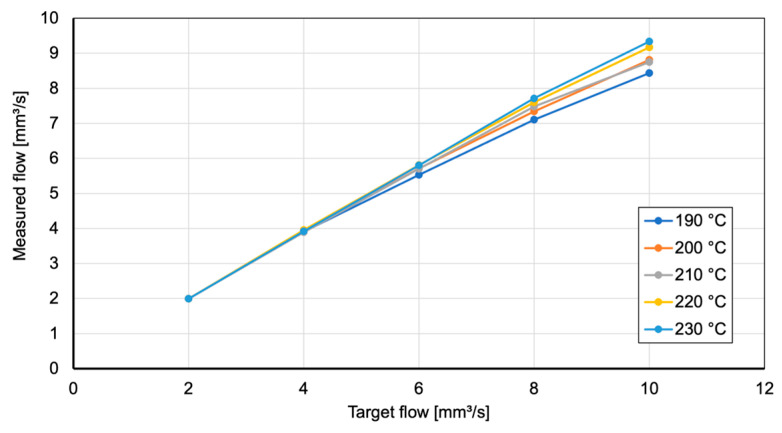
Comparison between target flow and measured flow for nozzle temperatures between 190 °C and 230 °C.

**Figure 11:**
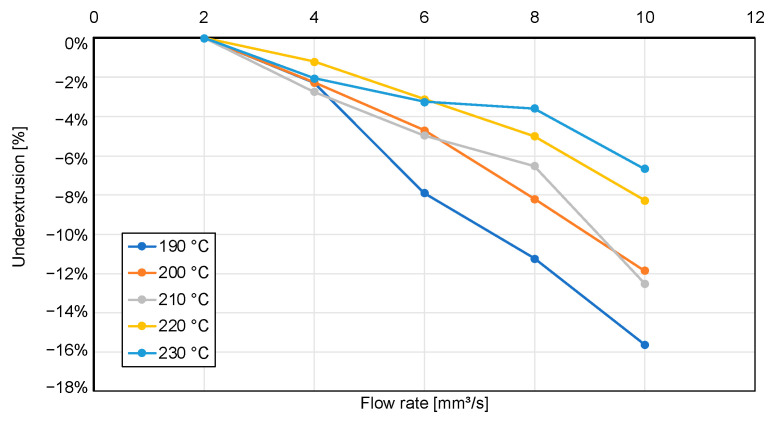
Calculation of underextrusion based on measurements shown in Figure 9.

Figure 10 underlines the findings from analyzing the ALE plot where higher nozzle temperature leads to higher flow. A flow value that more accurately matches the requested flow will lead to a higher weight accuracy. The impact of underextrusion, especially resulting from higher manufacturing and therefore extrusion speeds, can be seen in Figure 11. Here, underextrusion of 11.2% for the highest tested speed and the lowest tested nozzle temperature can be observed, noticeably impacting weight accuracy. 

**Figure 12:**
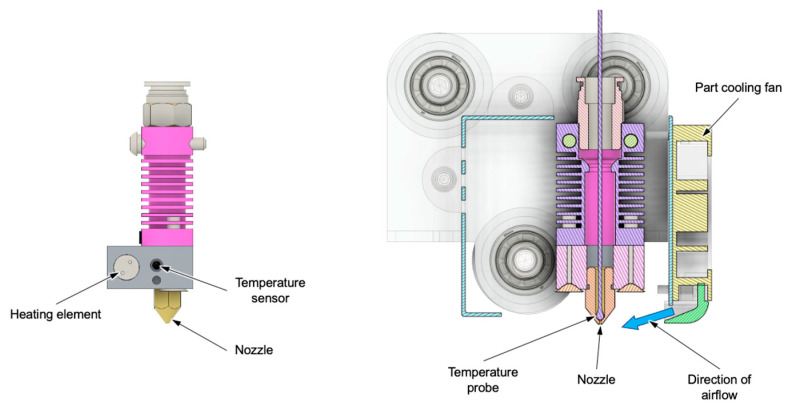
Mechanical assembly of plasticizing unit and location of part cooling fan.

Ideally, part cooling should not affect weight accuracy. However, the ALE score shows that high part cooling negatively impacts weight accuracy. Based on the previous investigations regarding the correlation between nozzle temperature and underextrusion, it is hypothesized that the airflow from the part cooling fan also negatively affects the nozzle temperature, leading to a second-degree effect on weight accuracy. However, during manufacturing, no deviation from the target temperature was recognized by the machine. It is possible that the effect is not detectable based on the temperature sensor’s location in the plasticizing unit, which is closer to the heating element than it is to the tip of the nozzle (see Figure 12, left). 

**Figure 13:**
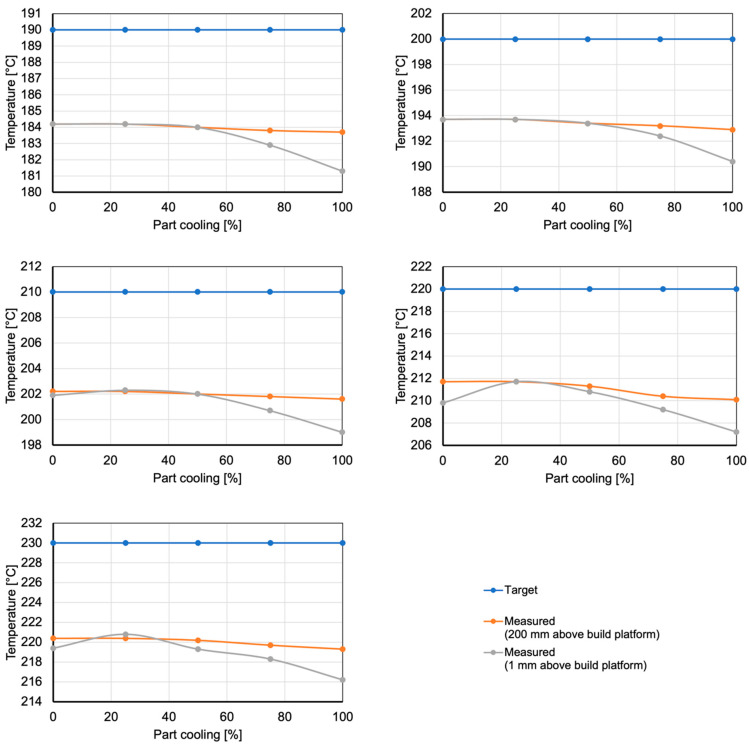
Measured nozzle temperature compared to target nozzle temperature for investigated steps of part cooling.

To investigate the hypothesis, a temperature probe is placed inside the nozzle (see Figure 12, right). Using this probe, temperatures are recorded for every combination of nozzle temperature and part cooling in a stationary trial. The test is performed for a z-position of 200 mm, meaning that the nozzle is located 200 mm above the build plate. To account for air reflections from either the build plate or the previous layer of a part being manufactured, the test is repeated for a z-position of 1 mm. The results are plotted in Figure 13. They show that, based on the locations of heating element and temperature sensor, the actual nozzle temperature is always between 5.8 °C to 10.6 °C below the requested temperature. 

Furthermore, they show a slight decrease in actual nozzle temperature for higher amounts of cooling at z = 200, as was expected. For z = 1, the temperature decrease is even more pronounced, resulting in a difference of up to 3.3 °C between 0% cooling and 100% cooling. Seeing as nozzle temperature is the major cause for underextrusion (Figure 11), this proves the hypothesis that part cooling effects nozzle temperature and is therefore a second-degree effect for weight accuracy.

### 4.6. Manufacturing Time

**Figure 14:**
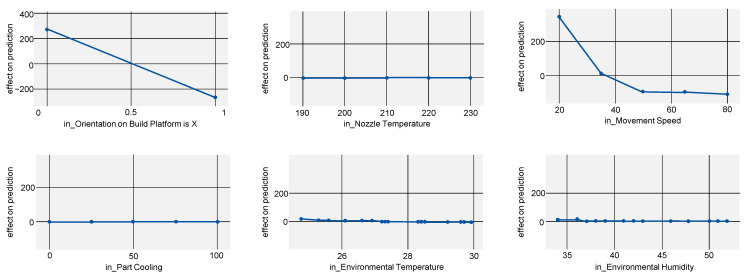
ALE indicating the effects of manufacturing time on the model prediction when varying all investigated process parameters.

With a coefficient of determination of $R^{2}=1.00$, the model for manufacturing time is the most precise. It shows that manufacturing time only depends on part orientation and movement speed, with the ALE plot (Figure 14) showing that a lying part is produced faster than a standing part. Other parameters, like nozzle temperature or part cooling, do not influence manufacturing time at all. 

Combining all individual investigations for each part property, it is noticeably via the PFI score that movement speed has little to no influence on the measured properties with the exception of manufacturing time. This suggests that in this set-up, movement speed should always be set as high as possible, resulting in minimal impact on part quality and reduced manufacturing time. However, it must be noted that actual machine movement does not only depend on configured movement speed in the slicer but also on the acceleration values of each axis as configured in the machine firmware. For the machine used in these trials, acceleration a is set to 500 mm/s^2^. Using this information, actual velocity v and distance traveled x can be obtained via integration:

(1) $$v=\int a\left(t\right)dt=a\cdot t+v_{0}$$

(2) $$x=\int v\left(t\right)dt=v\cdot t+x_{0}$$

with:

t: elapsed time
$v_{0}:$ initial velocity
$x_{0}:$ initial position

**Figure 15:**
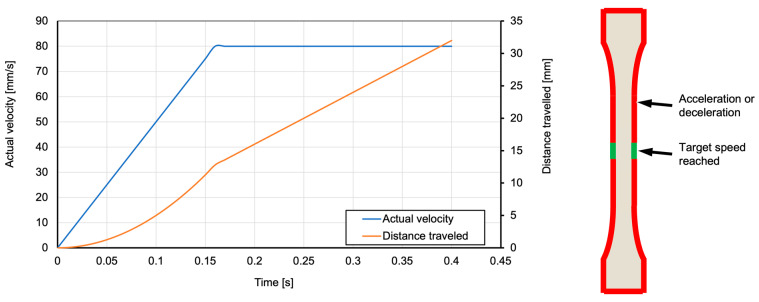
Actual velocity and distance traveled based on acceleration values (**left**), and areas of the test geometry where target speeds are reached at a requested speed of 80 mm/s (**right**).

Capping velocity at the highest set speed of 80 mm/s and plotting Equations (1) and (2) over 0.4 s (Figure 15, left) shows that the requested velocity is only reached after 0.16 s or 12.8 mm traveled. Given the small size of the test specimen, and also in with respect to deceleration, this means that the requested velocity is only reached for 4.4 mm of the 30 mm long testing zone (Figure 15, right). 

**Figure 16:**
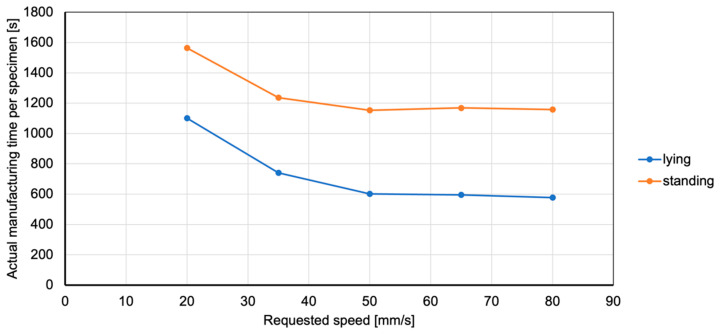
Actual manufacturing time does not decrease linearly with linear increase in requested manufacturing speed.

The effect is also observable when comparing manufacturing times. While a test specimen manufactured at 20 mm/s in lying orientation finishes after 1101 s on average, the same part takes 577 s at 80 mm/s. Even though manufacturing speed is quadrupled in this example, manufacturing time is only reduced by 47.6%, illustrating the significant impact of acceleration on small parts. Figure 16 plots manufacturing time for lying and standing test specimens in relation to the requested manufacturing speed. Both plots show an asymptotic progression with little to no actual manufacturing speed gained beyond 50 mm/s. The plot shows the same progression as the ALE score relating manufacturing speed and manufacturing time, further supporting the effortless interpretation of the effects via the feature importance toolkit.

Additionally, the ALE score relating manufacturing speed and weight accuracy decreases slightly after 65 mm/s, indicating that at higher speeds the machine is not capable of extruding the requested material in time, resulting in underfilled parts. This coincides with the analysis regarding weight accuracy and leads to the recommendation to set manufacturing speed no higher than 65 mm/s.

## 5. Summary and Outlook

This study investigates the use of the feature importance toolkit for analyzing an additive manufacturing process. By conducting a limited set of trials on a standardized test geometry and processing the results via the toolkit’s PFI and ALE scores, the manufacturing process can be understood thoroughly, and sweet spots for processing parameters, depending on the desired output, can be derived. It is shown via various experiments that progressions and interdependencies identified via the toolkit can be reproduced and validated and are rooted in physical correlations. To achieve this, a comparatively low amount of testing has to be performed in contrast to traditional approaches for characterizing a process in which multiple tests have to be conducted on various test specimens.

For the presented use-case of part quality achieved via FLM additive manufacturing, FI analysis clearly shows the importance of part orientation and therefore strand orientation in the part with regard to most measured quality factors. By comparing PFI scores, it becomes clear how dominant the orientation is in comparison to all other process parameters. Using the ALE scores, it is possible to analyze the remaining process parameters for their effects on part quality, like Young’s modulus, tensile strength, dimensional accuracy and weight accuracy. Here, the toolkit helps to unravel the interdependencies between nozzle temperature and part cooling as well as the machine’s limits in terms of extrusion amount and manufacturing speed. All identified correlations can be backed up by individual investigations. Since most FLM systems are based on the same core components and the system used for carrying out the investigations is one of the systems with the most units sold worldwide, the results are applicable to many extrusion-based AM systems. Furthermore, this study can be used as reference when investigating systems which differ significantly. Since the fundamental concepts still apply, approaches like transfer learning can be used to create detailed models about all FLM-systems while conducting significantly fewer trials. 

Based on this study’s results, the importance for process control in AM becomes apparent. Many of the identified shortcomings, like underextrusion or part cooling negatively affecting nozzle temperature, could be addressed by measuring relevant process values during the process and creating appropriate control loops. Since part orientation is identified as the most dominant factor for most quality aspects, optimized strand deposition methods should be derived to use individual strand directions in the most beneficial way.

## Figures and Tables

**Table 1:** Process parameters for generating trial data.

	Process Parameter	Unit	Value/Steps
*varied*	Nozzle Temperature	[°C]	190; 200; 210; 220; 230
	Manufacturing Speed	[mm/s]	20; 35; 50; 65; 80
	Part Cooling	[%]	0; 25; 50; 75; 100
	Part Orientation	[-]	Lying (y direction); standing (z direction)
*fixed*	Nozzle Diameter	[mm]	0.4
	Extrusion multiplier	[-]	1
	Extrusion width	[mm]	0.48
	Retraction	[mm]	4.9 (at 55 mm/s)
	Layer height	[mm]	0.2
	Top solid layers	[-]	3
	Bottom solid layers	[-]	3
	Perimeters	[-]	2
	Perimeter direction	[-]	Inside-out
	First layer height	[%]	150
	First layer width	[%]	120
	First layer speed	[%]	50
	Start points	[mm]	X = 0; Y = 500
	Skirt	[-]	1 Outline (only lying specimen)
	Brim	[-]	10 Outlines (only standing specimen)
	Infill	[-]	Rectilinear at 50% infill density; 20% overlap; 120% width; alternating between 45° and −45° for each layer
	Support	[-]	No support
	Heated bed	[°C]	50

**Table 2:** Testing results of manufactured samples. Trials are designated [temperature in °C]—[manufacturing speed in mm/s]—[part cooling in %]—[lying (L) or standing (S) orientation]. Each value represents the mean value of five test samples manufactured under identical conditions. * The samples for trial 210-20-50-S broke while being fixed in the tensile testing machine. Therefore, no mechanical results could be obtained for this trial.

Trial	Tensile Strength [MPa]	Young’s Modulus [MPa]	Elongation at Tensile Strength [%]	Manufacturing Time [s]	Dimensional Accuracy [%]	Weight Accuracy [%]
190-20-0-L	43.04	2317.2	2.48	1093.6	88.68	90.17
190-20-0-S	21.9	2179.2	1.14	1562.8	97.8	90.73
190-20-100-L	39.78	2210.8	2.44	1096.8	89.28	89.53
190-20-100-S	19.84	1985	1.104	1556.4	98.52	89
190-50-50-L	40.38	2288.2	2.38	600.6	86.72	88.91
190-50-50-S	23.26	2195	1.2	1151.6	97.4	90.2
190-80-0-L	40.16	2322	2.4	571.6	85.6	89.68
190-80-0-S	24.58	2243.8	1.22	1147.6	97.52	89.67
190-80-100-L	36.98	2124.8	2.4	572.2	90.44	88.15
190-80-100-S	17.36	2064.6	0.882	1157.4	98.88	88.99
200-50-50-L	40.66	2285.8	2.4	601.2	89.92	90.69
200-50-50-S	23.44	2172.2	1.17	1151	98.84	91.27
210-20-50-L	41.54	2341.2	2.34	1100.8	87.68	92.29
210-20-50-S	- *	- *	- *	1563.6	98.24	93.44
210-35-50-L	42.1	2343.6	2.38	740.8	87.2	92.13
210-35-50-S	22.38	2118.4	1.218	1235.6	97.88	92.33
210-50-0-L	42.28	2372.8	2.36	604	88.2	92.57
210-50-0-S	23.9	2167	1.232	1153.4	98.68	92.02
210-50-25-L	42.14	2388	2.34	603.2	87.56	92.06
210-50-25-S	24.2	2141.8	1.3	1153.2	98.68	91.89
210-50-50-L	41.32	2321.6	2.36	601.6	88.64	91.58
210-50-50-S	17.28	2099.2	0.88	1153	97.4	91.57
210-50-75-L	41.86	2342.2	2.42	602.4	88.64	91.98
210-50-75-S	19.12	2112.6	1	1153.2	97.6	91.14
210-50-100-L	41.04	2342.6	2.36	603.8	88.76	91.67
210-50-100-S	24.28	2122.4	1.26	1153.2	98.52	92.11
210-65-50-L	41.88	2317.2	2.42	594.8	87.96	91.33
210-65-50-S	22.56	2182.2	1.148	1168.6	98.36	92.15
210-80-50-L	40.8	2259.2	2.4	577.6	88.64	91.54
210-80-50-S	26.66	2150.6	1.44	1153.6	96.56	92.28
220-50-50-L	42.44	2427.2	2.36	601.4	87.4	92.33
220-50-50-S	19.54	2134	1.008	1152.4	98.92	92.38
230-20-0-L	45.08	2436.2	2.46	1106.2	87.24	94.09
230-20-0-S	24.74	2293.6	1.244	1561.8	98.56	95.41
230-20-100-L	40.9	2208.4	2.4	1106	87.44	93.25
230-20-100-S	22.88	2186.4	1.16	1573	97.92	95.18
230-50-50-L	43.36	2464.6	2.36	601.8	87.52	93.56
230-50-50-S	12.9	2137.4	0.612	1152.6	98.76	93.52
230-80-0-L	43.6	2407.2	2.46	579.8	86.6	93.21
230-80-0-S	30.26	2285.6	1.56	1164	99.2	94.35
230-80-100-L	41.24	2247.4	2.42	581.8	89.76	92.56
230-80-100-S	22.66	2113.2	1.2	1166.6	96.08	94.08

**Table 3:** Hyperparameter optimization parameters.

Model Type	Maximal Depth of Tree	Minimal Samples per Split	Minimal Samples per Leaf	Number of Estimators
DecisionTreeRegressor	1, 2, 3	2, 5, 10	1, 2, 5, 10	-
ExtraTreeRegressor	1, 2, 3	2, 5, 10	1, 2, 5, 10	-
RandomForestRegressor	1, 2, 3	2, 5, 10	1, 2, 5, 10	10, 20, 50, 100
GradientBoostingRegressor	1, 2, 3	2, 5, 10	1, 2, 5, 10	10, 20, 50, 100

**Table 4:** Best performing models of each model type after hyperparameter optimization in terms of *R*^2^ score. For the best performing model, the values of the hyperparameters are given.

Target	Decision Tree Regressor	Extra Tree Regressor	Random Forest Regressor	Gradient Boosting Regressor	Best Model Type	Maximal Depth	Minimal Samples per Split	Minimal Samples per Leaf	Number of Estimators
**Young’s** **modulus**	0.489	0.469	0.533	0.558	**Gradient Boosting Regressor**	3	5	2	20
**Tensile strength**	0.936	0.913	0.939	0.930	**Random Forest Regressor**	3	1	2	50
**Elongation at tensile strength**	0.950	0.929	0.948	0.939	**Decision Tree** **Regressor**	3	1	2	n.a.
**Dimensional accuracy**	0.931	0.924	0.939	0.943	**Gradient Boosting Regressor**	3	1	5	50
**Weight** **accuracy**	0.438	0.461	0.394	0.424	**Extra Tree** **Regressor**	3	1	10	n.a.
**Manufacturing time**	0.999	0.994	0.999	1.000	**Gradient Boosting Regressor**	3	1	2	100

## Data Availability

The data presented in this study are available on request from the corresponding author.
